# Effect of mineral-vitamin premix supplementation on behavioral, performance, hormonal, oxidative stress, and serum biochemical profiles on rutting male *Camelus dromedarius* in Egypt

**DOI:** 10.3389/fvets.2023.1221830

**Published:** 2023-10-20

**Authors:** Nani Nasreldin, Ramadan D. EL-Shoukary, Ghada S. E. Abdel-Raheem, Heba S. Gharib, František Zigo, Zuzana Farkašová, Ibrahim F. Rehan, Waleed Senosy

**Affiliations:** ^1^Department of Pathology and Clinical Pathology, Faculty of Veterinary Medicine, New Valley University, El-Kharga, Egypt; ^2^Department of Development of Animal Wealth, Faculty of Veterinary Medicine, New Valley University, El-Kharga, Egypt; ^3^Departments of Nutrition and Clinical Nutrition, Faculty of Veterinary Medicine, Assiut University, Asyut, Egypt; ^4^Department of Behaviour and Management of Animal, Poultry and Aquatic, Faculty of Veterinary Medicine, Zagazig University, Zagazig, Egypt; ^5^Department of Nutrition and Animal Husbandry, University of Veterinary Medicine and Pharmacy, Košice, Komenského, Slovakia; ^6^Department of Husbandry and Development of Animal Wealth, Faculty of Veterinary Medicine, Menoufia University, Shebin Alkom, Menoufia, Egypt; ^7^Department of Pathobiochemistry, Faculty of Pharmacy, Meijo University, Nagoya-shi, Japan; ^8^Department of Theriogenology, Faculty of Veterinary Medicine, New Valley University, El-Kharga, Egypt

**Keywords:** rut, *Camelus dromedaries*, behavior, oxidative stress, mineral-vitamin premix

## Abstract

**Introduction:**

The rutting period imposes a stressful condition on male camels, which results in elevated serum cortisol levels and alterations in their sexual behavior. Therefore, the current work was carried out to investigate the effect of mineral-vitamin premix supplementation on behavior, reproductive performance, hormones, serum oxidative stress profile, and other serum biochemical parameters of *Camelus dromedarius* during the breeding season.

**Methods:**

Fourteen mature, fertile male *Camelus dromedarius* were divided into two groups, a control group (*n* = 7) and a mineral-vitamin premix group (*n* = 7). The present study lasted for 95 days during the rutting period (1st February to 5th May). Each camel in the premix group received a daily diet of 50 g of mineral-vitamin premix throughout the whole rutting period, during which the frequencies and durations of the following behaviors: maintenance, posture, aggressiveness, and sexual activity were collected every 20 min. At the end of the study, blood samples were collected.

**Results:**

Results revealed that the premix group showed higher (*P* < 0.05) maintenance (feeding and rumination), standing, and overall sexual desire-related behavior frequency, besides more times (*P* < 0.001) for rumination, standing, walking, and lying while showing lower (*P* < 0.001) frequencies of overall aggressive behaviors than the control group. The serum concentration of malondialdehyde, nitric oxide, cortisol, blood glucose, and urea evidenced a significant decrease in the premix group compared with the control one, while significantly elevated levels of reduced glutathione, testosterone, total antioxidant capacity, triiodothyronine, and thyroxin, total protein, albumin, globulin, calcium, phosphorus, potassium, and magnesium were recorded in the premix group in comparison with the control.

**Conclusion:**

It could be concluded that daily dietary supplementation of 50 g of mineral-vitamin premix to male camels during the breeding season is necessary to overcome the oxidative stress and serum cortisol concentration with a subsequent decrease in aggressive behavior and improvement to testosterone level in blood, body condition score and body weight gain.

## Introduction

Approximately 75% of the *Camelus dromedarius* population is raised in arid and semi-arid regions, including Egypt, due to the unique physiological characteristics that enable them to survive and perform in such harsh environments (1). Unfortunately, these regions are deficient in mineral elements, which adversely affect animal production and reproduction (2). In addition, due to climatic changes and global warming, camel farmers changed their production systems from those based on herd mobility to primarily settled and semi-intensive systems. Moreover, in the more intensive systems, camel feeding becomes progressively dependent on supplements as a means to provide the nutrient requirements. Furthermore, the feeding system of camels changed from a highly diversified diet (with high variability in nutritive value and grazed ecosystems) to a standard diet (typically alfalfa, occasionally barley, and concentrates). Such diets do not necessarily cover the nutrient requirements, including trace minerals (3).

The rutting period implies the breeding season of male *Camelus dromedarius*, which extends for 120–180 days in Egypt (4). It imposes a stressful condition on male camels, leading to a change in the feeding behavioral pattern of camels, including partial to complete loss of appetite (off food) (5, 6), and the body condition of camels deteriorates during this period accompanied by a reduction in body weight (7). Moreover, camels exhibit aggressiveness toward other males and humans (8). This aggressiveness is simultaneous with an elevation in serum cortisol concentration (9), which drives Camelidae farmers to tie male camels with ropes in single stalls (10, 11). More than that, the elevated cortisol concentration negatively affects testosterone production by Leydig cells (10), with subsequent alterations in their sexual behavior (9, 10). Furthermore, the regions of raising camels (arid and semiarid), the change in the rearing system, and the rutting period may all be associated with a deficiency in mineral or vitamin elements, which adversely affect the animal's production and reproduction, and camels may suffer from the growth retardation of newborns, low feed efficiency, anemia, poor fertility, poor reproduction, and many other metabolic disorders (2, 3). In this regard, trace minerals are crucial for animal health and productivity due to their multiple functions, roles, and activities within the animal body which are affected by many factors such as genes, age, and different requirements for maintenance and growth, as well as the level of production and reproduction (2, 3, 12). In addition, an animal's trace mineral status has an important role in different physiological stages, especially throughout the stress periods and during hard exercise, so its requirements can be increased to meet the immune and metabolic stress demands (12, 13). Any deficiency in trace minerals may result in a variety of pathological problems and metabolic defects (3). Therefore, the strategic use of feed additives has the potential to increase the efficiency of feed and animal production. Feed efficiency is a repercussion of health, nutrition quality, management, and reproductive performance (14), and camels must be supplied with all essential nutrients despite the hostile environment, same as other animals (2).

Finally, as a result of the steady increase in human population numbers and the insufficient food production in Africa and parts of Asia, it is compulsory to develop suitable systems for livestock production, accompanied by the optimal and effective utilization of available and untapped native resources (15). In this regard, camels could be a future target due to being regarded as one of the most important meat-producing animals in Egypt. Moreover, the use of camels as a source of meat, milk, and work has been highly elevated in recent years (15). Therefore, this study was conducted to investigate the impact of the addition of mineral-vitamin premix to the ration of mature male *Camelus dromedarius* during the rutting period in Egypt to overcome the negative impact of rutting season on the behavior (walking, standing, lying, rumination, and sexual activity), performance (body condition score, body weight, and body weight gain), serum oxidative stress profile [total antioxidant capacity (TAC), malondialdehyde (MDA), nitric oxide (NO), and reduced glutathione (GSH)], hormone levels [testosterone, cortisol, triiodothyronine (T3), and thyroxin (T4)], and other serum biochemical parameters [total protein (TP), albumin, globulin, A/G ratio, blood glucose, creatinine, urea, calcium (Ca+2), phosphorus, potassium (K+), and magnesium].

## Materials and methods

### Ethical approval

The protocol of this experiment was approved by Zagazig University's institutional animal care and use committee (ZU-IACUC) under the number (ZU-IACUC\2\F\80\2023).

### Animals and management

Fourteen mature fertile male *Camelus dromedarius* aged 5–8 years, that reached puberty at 3–4 years (16), weighed 400–450 kg, and had a body condition score of 3.5 ± 0.35 standard error mean (SEM) (17), were used in the current study. This field study was conducted on *Camelus dromedarius* that were owned by camel owners in Bani-Adi village, Assiut governorate (latitude 24° 48′N and longitude 46° 31′E) in Egypt. This study lasted for 95 days during the rutting season (1 February-5 May). Semi-covered areas with sand floors (15 m^2^/camel) were used as housing (18).

*Camelus dromedarius* has been fed a commercial diet (Abo Donkol Feeds, Egypt) with a constant quantity and quality of feed during the experiment period with free access to fresh water. The quantity was approximately 3% of their live weight in dry matter, while the quality was 40% concentrate (11% protein) and 60% hay. The recommended feeding schedule was 8–9 a.m. concentrate, 11 a.m.−12 p.m. hay, and 4–5 p.m. concentrate.

### Design of the study

**Figure 1:**
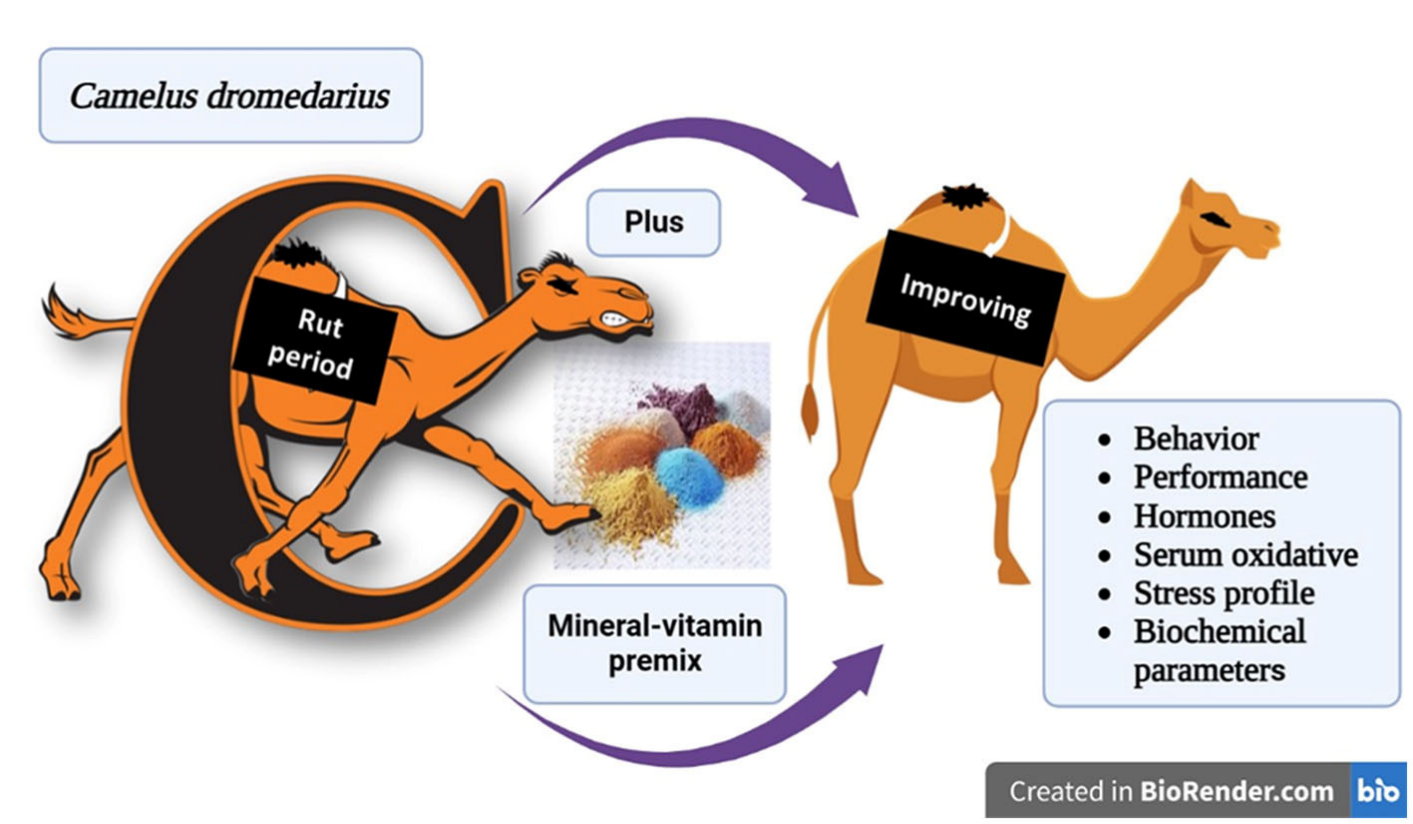
The schematic cartoon of the study. It is designed by BioRender.com.

Fourteen male dromedary camels were used (*n* = 7 per group): the control group was fed the basal diet only, and the premix group received the control diet plus 50 g of mineral-vitamin premix per camel daily throughout the whole rutting period. The schematic cartoon of this study is illustrated in Figure 1. All the chemical constituents of the mineral and vitamin premix are listed in Table 1. The study period, from 1 February to 5 May (95 days), was divided into two periods. The first period was the adaptation period which extended from 1 to 28 February (4 weeks), where on 2, 9, 16, and 23 February the animals were watched for 1 h twice weekly (Saturday and Tuesday) in the morning (8:20–8:40), midafternoon (12:20–12:40), and afternoon (3:20–3:40) to give the camels time to adapt to unfamiliar human and/ or camera presence. Also, the management system of camels was similar in all groups during the study, and getting rid of any abnormal health conditions such as cough, fever, weakness, or any abnormal behavior camel may display (such as a lame camel or biting or kicking). The second period was the rutting period, which lasted from 1 March to 5 May (66 days, nearly 10 weeks), which is considered the breeding season of camels in Egypt (18).

**Table 1:** The chemical constituents of the mineral and vitamin premix.

**Ingredients**	**Amount per 50 g**
Vitamin (Vit.) A (IU)	7,619
Vit. D_3_ (IU)	2,619
Vit. E (mg)	27.15
Vit. B_1_ (mg)	2.4
Vit. B_2_ (mg)	0.95
Vit. B_6_ (mg)	2.85
Vit. B_12_ (mg)	14.3
Vit. C (mg)	2.15
Vit. K_3_ (mg)	2.4
Nicotinamide (mg)	14.3
Methionine (mg)	14.3
Calcium pantothenate (mg)	3.2
Folic acid (mg)	1.45
Choline chloride (mg)	4.75
Biotin (mg)	2.85
Magnesium sulfate (mg)	288.1
Selenium (mg)	1.35
Zinc sulfate (mg)	264.3
Copper sulfate (mg)	139.3
Cobalt sulfate (mg)	2.4
Manganese (mg)	238.7
Iron sulfate (mg)	369.05
Iodine (mg)	1.45
Sodium chloride (g)	7.05
Potassium iodate (ppm)	11.9

### Behavioral variables observation

Totally 280 h of camels' behaviors (140 h per group) were recorded (1 h twice a week (Saturday and Tuesday) as 20 min in the morning (8:20–8:40), midafternoon (12:20–12:40), and afternoon (3:20–3:40) for 10 weeks, which were analyzed using the focal sample technique [3 times per day\2 days (Saturday and Tuesday)\week = 1 h × 2 days × 10 weeks × 7 camels = 140 h per each group] (19). The behaviors were recorded visually using a notebook for recording behavior and a stopwatch. The posture (including standing, lying, and walking), maintenance (including feeding, drinking, and rumination), aggressive behavior (including biting, kicking, and fighting), and sexual behavior (including teeth grinding, blathering, gulla extrusion, tail flapping, open legs, yawning, poll gland secretion, neck rubbing, and urination) durations and their frequencies per 20 min were recorded according to the ethogram (15).

### Trough leftovers score

Visual observation was done daily after 2 h of feeding for the scoring of trough leftovers. The scores were assessed on a scale from 1 to 5, according to Stadler et al. (20).

### Evaluation of average body weight and body weight gain

The average of initial and final body weights was weighted by kilograms (kg), and the average weight gain was calculated by the difference between them.

### Evaluation of the body condition score

Body condition score was evaluated *via* visual assessment from an interval space of 2–3 m far from the camel, while hump size was assessed by an approximate determination of the dorsal line proportion occupied by the body structure as illustrated by Robinson (21).

Body condition score represented by 0–5 which refers to the flank and rib regions, and another score of 0–5 refers to the hindquarters, which is illustrated by Faye et al. (17) and explained in tables by Iglesias et al. (22).

The average score will be the final value for the body condition of each individual (23).

### Blood sampling

At the end of the rutting period, the blood specimen was taken from the jugular vein and placed into plain vacuum tubes. Then, the tubes were left for 20 min in an inclined position at room temperature. After that, the tubes were kept in the fridge to evade glycolysis and until there was complete blood clot retraction. Following that, the samples were put in the centrifuge for 10 min at 3,000 rpm (1,107 × *g*) for the separation of the clear serum, which was carefully collected in Eppendorf tubes and stored at −80°C until hormonal and biochemical analysis.

### Oxidative stress analysis

Oxidative stress variables, including serum malondialdehyde (MDA), total antioxidant capacity (TAC), reduced glutathione (GSH), and nitric oxide (NO), were analyzed *via* the colorimetric method using commercial kits (Biodiagnostic, Egypt). Oxidative stress variables were assayed using a semi-automatic photometer (5,010 V5+, photometer, RIELE Co., Germany) according to the manufacturer's instructions.

### Hormonal analysis

Serum concentrations of cortisol, triiodothyronine (T3), thyroxin (T4), and testosterone were investigated using commercial ELISA kits (Accu Bind Monobind Inc., Lake Forest, United States). The ELISA reader was used (ChroMate, Model 4300 microplate reader, FL, United States) at 450 nm.

### Serum biochemical analysis

Serum biochemical parameters, including blood glucose, total protein (SPINREACT, Spain), urea (Diamond, Egypt), creatinine, albumin, calcium phosphorus, magnesium, and potassium (Human Co., Germany), were investigated *via* the colorimetric method using the commercial kits. Globulin levels and the albumin/globulin (A/G) ratio were calculated (24). All parameters were determined using a semi-automatic photometer (5010 V5+, photometer, RIELE Co., Germany) according to the manufacturer's instructions.

### Statistical analysis

All obtained data variables were analyzed using independent-sample *t-*tests using the SPSS software program version 23.0 (25). The obtained result was expressed as mean ± standard error (SE), and *P* < 0.05 was assumed to reflect statistical significance.

## Results

### Behavioral variables

**Figure 2:**
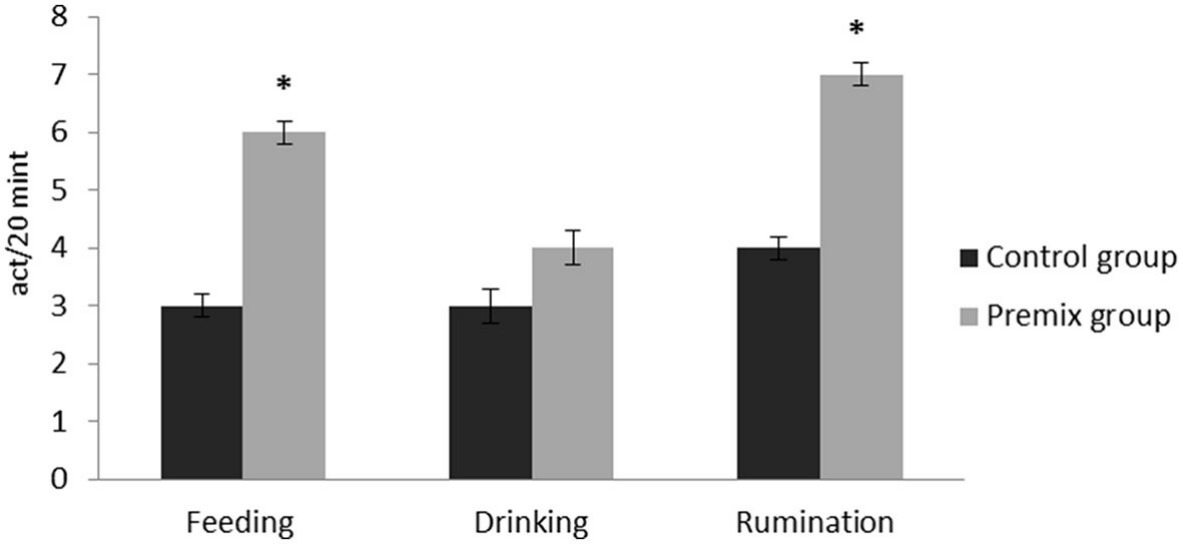
Effect of mineral-vitamin premix supplementation on maintenance frequency behaviors of male *Camelus dromedarius* during the rut season. *Significance (*P < 0.05), compared with the control group using an independent-sample *t-*test.

**Figure 3:**
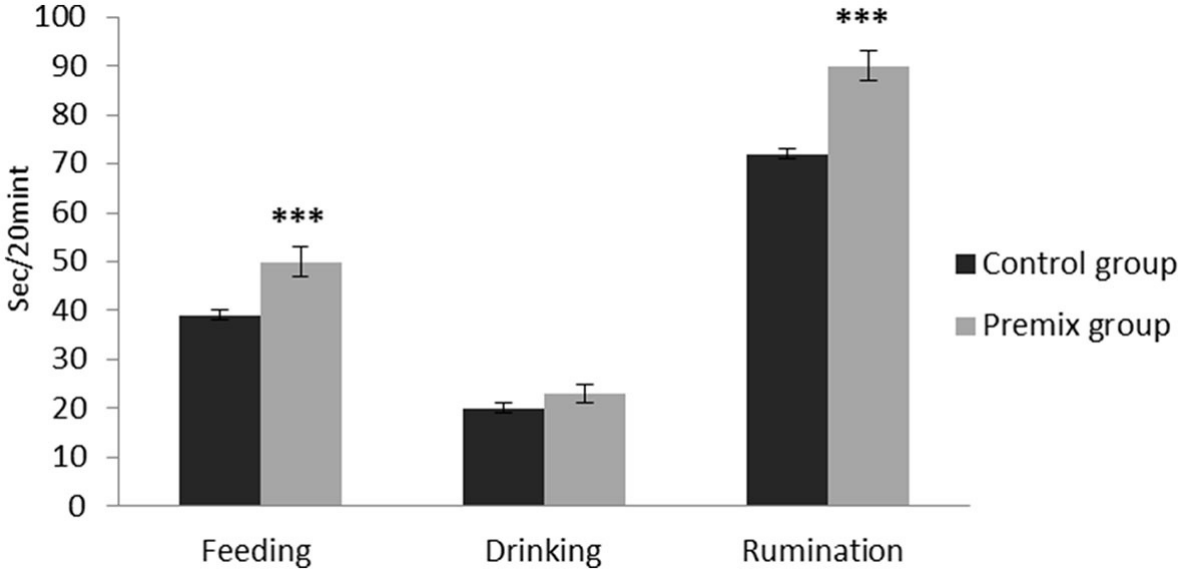
Effect of mineral-vitamin premix supplementation on maintenance duration behaviors of male *Camelus dromedarius* during the rut season. *Significance (***P < 0.001), compared with the control group using an independent-sample *t-*test.

**Figure 4:**
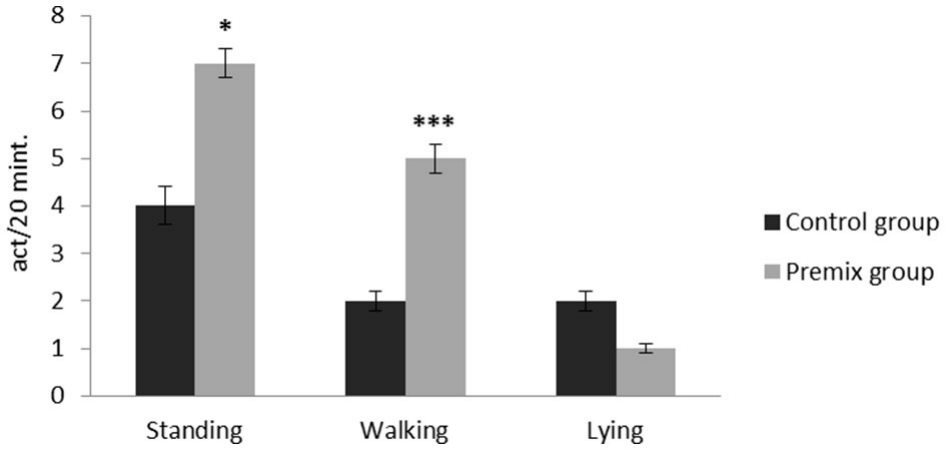
Effect of mineral-vitamin premix supplementation on posture frequency behaviors of male *Camelus dromedarius* during the rut season. *Significance (*P < 0.05, ***P < 0.001), compared with the control group using an independent-sample *t-*test.

**Figure 5:**
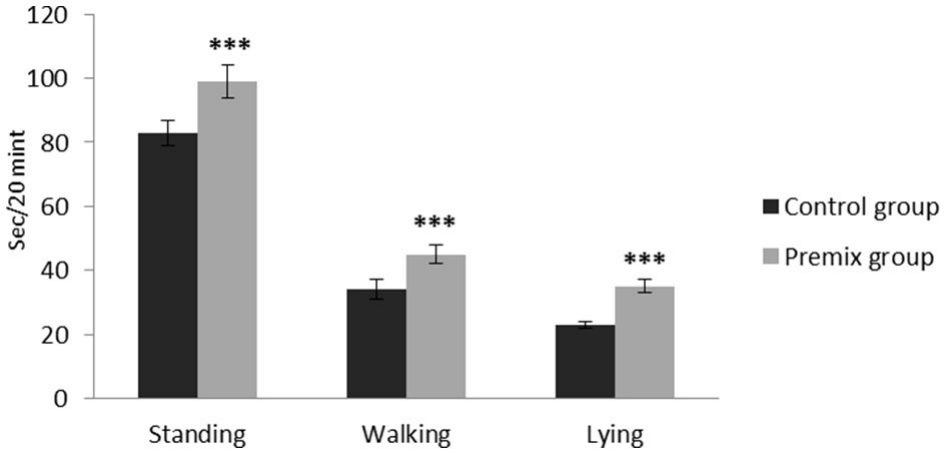
Effect of mineral-vitamin premix supplementation on posture duration behaviors of male *Camelus dromedarius* during the rut season. *Significance (***P < 0.001), compared with the control group using an independent-sample *t-*test.

**Figure 6:**
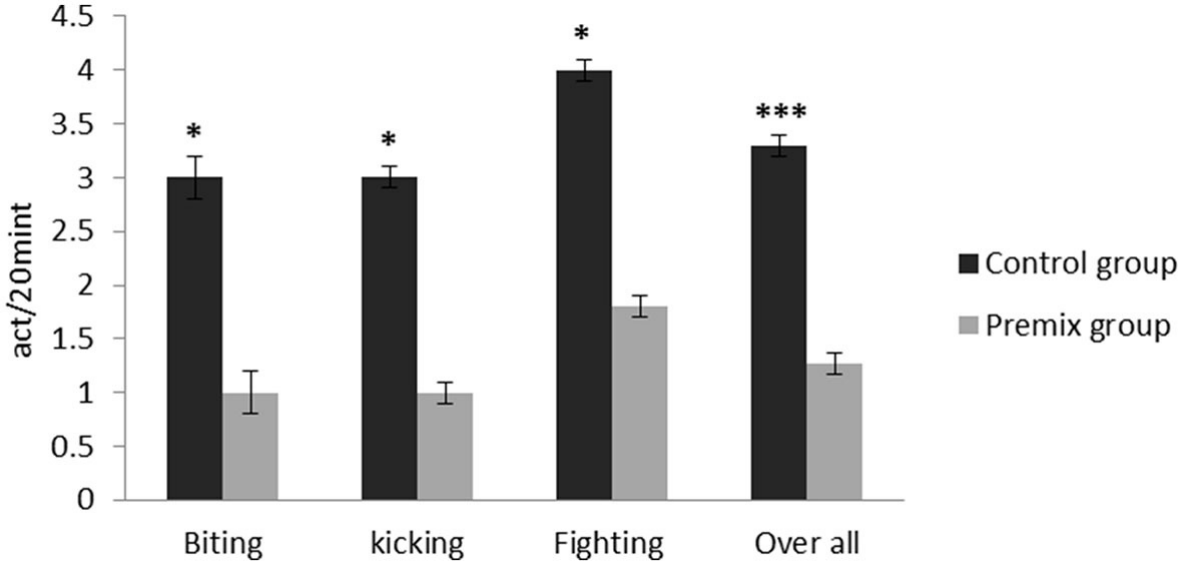
Effect of mineral-vitamin premix supplementation on aggressive behaviors of male *Camelus dromedarius* during the rut season. *Significance (*P < 0.05, ***P < 0.001), compared with the control group using an independent-sample *t-*test.

**Figure 7:**
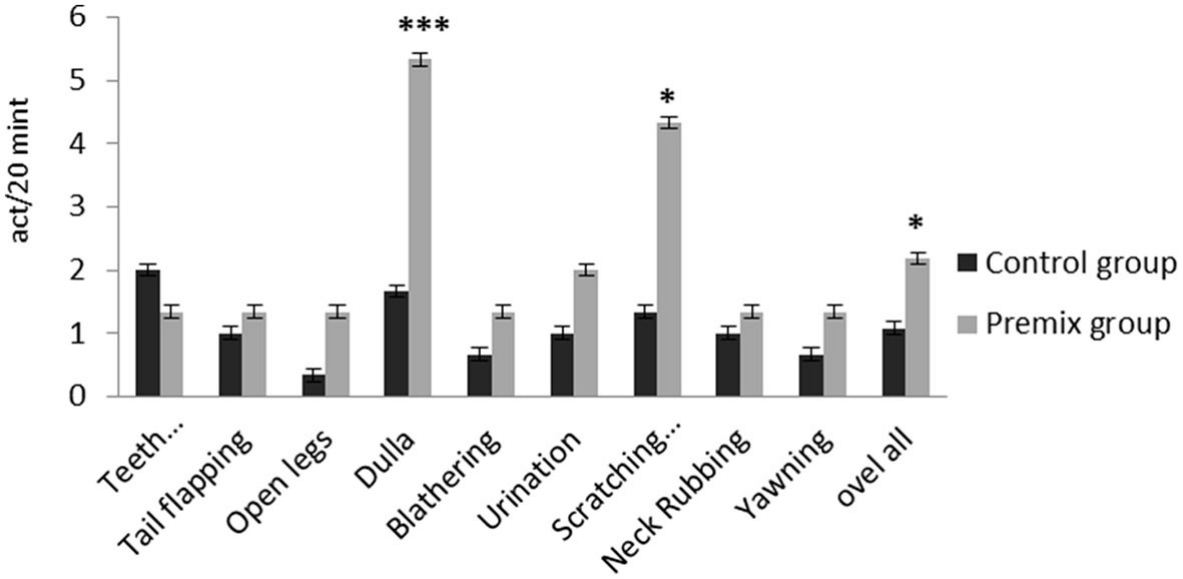
Effect of mineral-vitamin premix supplementation on sexual behaviors of male *Camelus dromedarius* during the rut season. *Significance (*P < 0.05, ***P < 0.001), compared with the control group using an independent-sample *t-*test.

The effects of mineral-vitamin premix supplementation on maintenance behavior (frequency and duration), posture, and aggressive and sexual behaviors of male *Camelus dromedarius* during the rutting season are shown in Figures 2–7. Mineral-vitamin premix supplementation had a significant effect on maintenance and posture behaviors. The premix group showed higher (*P* < 0.05) frequencies of maintenance (feeding and rumination, Figures 2, 3) and posture (standing and walking, Figures 4, 5) as compared with the control group. The time consumed for feeding, rumination, and walking behaviors showed a significant increase (*P* < 0.001) in the premix group in comparison with the control group. Mineral-vitamin premix supplementation significantly affected the sexual and aggressive behaviors of male dromedaries, where the premix group expressed a higher (*P* < 0.05) frequency of some sexual desire-related behaviors, including extruding gulla and poll gland secretion. On the other hand, there was a significant decrease in the frequency of aggressive behaviors compared with the control group, as shown in Figures 6, 7.

### Scoring of trough leftovers, body weight, body gain, and body condition

The scoring of trough leftovers, average final body weight, body gain, and body condition score were significantly elevated in the premix-treated group in comparison with the control one (Table 2).

**Table 2:** Effect of mineral-vitamin premix supplementation on the scoring of trough leftovers, body weight, body gain, and body condition score (mean ± standard error) of male *Camelus dromedarius* during rut season (*n* = 7/group).

	**Control group**	**Premix group**
Scoring of trough leftovers	3.14 ± 0.29	2.42 ± 0.20^*^
Body condition score	2.57 ± 0.20	3.29 ± 0.14^*^
Average initial body weight (kg)	404.86 ± 2.13	408.14 ± 2.28
Average final body weight (kg)	485.00 ± 6.47	515.00 ± 6.89^***^
Average body weight gain (kg)	80.14 ± 6.16	106.87 ± 6.66^*^

Significance (^*^P < 0.05 and ^***^P < 0.001), compared with the control group using an independent-sample *t-*test. Kg: Kilogram.

### Oxidative stress variables

Both serum MDA and NO evidenced a significant decrease in the premix-treated group as compared with the control group. On the other hand, both serum GSH and TAC were significantly increased (Table 3) in the premix group compared with the control group.

**Table 3:** Effect of mineral-vitamin premix supplementation on oxidative stress profiles (mean ± standard error) of male *Camelus dromedarius* during rut season (*n* = 7/group).

**Oxidative stress parameter**	**Control group**	**Premix group**
Malondialdehyde (nmol/ml)	2.34 ± 0.10	1.75 ± 0.06^**^
Nitric oxide (μmol/L)	79.56 ± 1.17	36.37 ± 0.66^***^
Reduced glutathione (mg/dl)	1.23 ± 0.07	1.90 ± 0.12^**^
Total antioxidant capacity (mM/L)	1.70 ± 0.05	2.2 ± 0.08^***^

^*^Significance (^**^P < 0.01, ^***^P < 0.001), compared with the control group using an independent-sample *t-*test.

### Hormonal variables

The premix group had a significant increase in serum testosterone, T_3_, and T_4_ levels. However, it had a significantly lower serum cortisol level than the control (Table 4).

**Table 4:** Effect of mineral-vitamin premix supplementation on hormonal profiles (mean ± standard error) of male *Camelus dromedarius* during rut season (*n* = 7/group).

**Hormonal profiles**	**Control group**	**Premix group**
Cortisol (ng/ml)	552.9 ± 14.1	435.7 ± 10.7^***^
Testosterone (ng/ml)	1.33 ± 0.097	1.93 ± 0.05^***^
Triiodothyronine (ng/ml)	2.74 ± 0.08	3.19 ± 0.07^**^
Thyroxin (μg/dl)	3.19 ± 0.06	3.74 ± 0.0997^**^

^*^Significance (^**^P < 0.01, ^***^P < 0.001), compared with the control group using an independent-sample *t-*test.

### Serum biochemical variables

There was a significant increase (*P* < 0.05) in serum TP, albumin, Ca^+2^, P^+3^, magnesium, Mg^+2^, and K^+^ in the premix group compared with the control (Table 5). Meanwhile, a significant decrease (*P* < 0.05) was recorded in blood glucose and urea in the premix group compared with the control group (Table 5).

**Table 5:** Effect of mineral-vitamin premix supplementation on serum biochemical profiles (mean ± standard error) of male *Camelus dromedarius* during rut season (*n* = 7/group).

**Serum biochemical profiles**	**Control group**	**Premix group**
Total protein (g/dl)	6.14 ± 0.09	6.87 ± 0.11^***^
Albumin (g/dl)	3.79 ± 0.10	4.4 ± 0.08^**^
Globulin (g/dl)	2.36 ± 0.01	2.47 ± 0.12
Albumin/globulin ratio	1.63 ± 0.11	1.82 ± 0.12
Blood glucose (mg/dl)	91.46 ± 1.66	79.33 ± 0.71^***^
Urea (mg/dl)	17.67 ± 0.31	12.40 ± 0.22^***^
Creatinine (mg/dl)	1.53 ± 0.06	1.54 ± 0.04
Calcium (mg/dl)	2.10 ± 0.08	2.61 ± 0.09^**^
Phosphorus (mg/dl)	1.80 ± 0.04	2.30 ± 0.05^***^
Magnesium (mg/dl)	0.88 ± 0.07	0.98 ± 0.03
Potassium (mmol/L)	3.24 ± 0.06	3.80 ± 0.08^***^

^*^Significance (^**^P < 0.01, ^***^P < 0.001), compared with the control group using an independent-sample *t-*test.

## Discussion

The body condition score of an animal denotes the quantity of fat and muscle reserves that are available for maintenance, reproduction, and production, and it is regarded as a main tool for livestock managers or producers to optimize the feeding program, reproduction, production (milk and meat), and welfare of the animals. Scoring is based on determining the amount of fat and muscles over and around the vertebrae (26). In addition, BCS is the best simple indicator of body fat reserves and can be used by the animal itself in periods of high energy demands, various stresses, or undernutrition conditions, making it a widely accepted indicator of post-nutritional status (27). In this study, the BCS in both the control and premix groups revealed a reduction in the body condition score compared with that at the beginning of the study before the entrance of the camels in the rutting period. This may be due to the fact that the rutting period decreases feed intake, resulting in a markedly tucked-up abdomen, a gradual decrease in hump size (28), and rutting males losing their body condition (7).

The obtained data revealed a significant increase in feeding, rumination behaviors (frequency and duration), final body weight, body weight gain, and body condition score, while there was a significant decrease in the leftover score in the premix group as compared with the control. This result matches that of Faye et al. (29) who recorded a slight improvement in the feed intake of young camels (<2 years old) with copper mineral supplementation. Such improvement is due to the Zn element contained in the mineral-vitamin premix, which has a positive effect on appetite, causing increased feed intake (30, 31).

Moreover, feed efficiency and average daily gain had an irreversible relation to protein and energy levels in the diet (32). Furthermore, the level of energy is significantly related to the average daily feed intake (feeding behavior as an indicator), and the improvement of nutrient digestibility by the mineral-vitamin premix led to optimization of the microbial ecosystem of the rumen with subsequent enhancement of the productive performance, besides its involvement in the enzymatic system formation (33). More than that, mineral-vitamin premix plays a significant role in numerous metabolic processes, production (biological functions), and regulation in the body (30, 34). In the same line, mineral-vitamin premix supplementation had a positive effect on growth performance in buffalo calves (35) and growing male camels (36), where mineral supplementation such as zinc (Zn), copper (Cu), manganese (Mn), and selenium (Se) caused an increase in average daily gain and improved growth rates (37–40). Moreover, the ruminants' productive performance can be affected directly and indirectly by Zn, Se, Cu, iodine (I), and Mn levels as trace minerals due to their role in many organic functions, e.g., thyroid hormone activity, enzymatic, metabolic, and productive roles (30, 34).

In addition, data in the current study showed significantly increased activity in standing and walking in the premix group as compared with the control group, which may be a result of displaced animals waiting for access to the feed (41) and camelids always moving in a single file (42). Moreover, lying behavior is considered a good indicator to determine animal comfort and to provide important data about animal-environment interaction, such as the duration of lying. Such measures have been identified as significant measures used to assess stall comfort and welfare status (43, 44). According to the data of the current experiment, there was a significant difference in lying time between the premix group and the control group, which may be due to the relationships between eating and rumination (45).

Aggressive behavior is considered a functional form of social behavior that can be seen in nearly all animals, and it has many functions, such as protecting the home range and its resources against intruders and/or maintaining the flock's social hierarchy (46).

Rut season in male camels constitutes a stress period that is accompanied by a general state of increased irritability and aggressiveness, which is associated with an elevated serum cortisol level (9, 47). Due to this aggressiveness, camel raisers resort to tying male camels with ropes in single stalls, which constitutes additional stress on the camels (10). Furthermore, aggressive behavior is highly harmful, and it triggers a robust stress response in both the winner and the loser, where both winning and losing (offense and defense) provoke a differential stress response in terms of the magnitude of the responses and the neuroendocrine stress systems, leading to the vulnerability for stress pathology (46). In addition, this study also observed that to take blood samples, animals had to be roped in at the kneeling position, and due to their aggressive behavior, which could be very harmful when taking blood samples, camels were subjected to strict physical restraint. This physical restraint alone exerts acute stress on the animal, causing an outpouring of adrenocorticotropic hormone (ACTH), which in turn causes the adrenal cortex to increase its secretion of glucocorticoids, including cortisol (48–50). On the same line, the traditional or manual blood sampling method has its own restrictions and cannot possibly be used due to male camel aggression during this period. The same restrictions can also lead to stress reactions even in animals accustomed to the given procedure (51) that result in increased levels of cortisol due to the activation of the hypothalamic stimulation-pituitary-adrenal axis during this sampling (52). Moreover, Emeash et al. (53) assessed the stress resulting from the transportation of dromedary camel (*Camelus dromedarius*) and recorded that cortisol levels significantly increased (17.21 ± 2.96 μg/dl = 172.1 ± 29.6 ng/ml) in camels that were transported by truck (they traveled approximately 150 km, taking approximately 2–3 h) between mountains as compared with control (3.35 ± 0.38 μg/dl = 33.5 ± 3.8 ng/ml).

In this regard, the results of the current study showed a significant decrease in overall aggressive behavior frequency in the premix group in comparison with the control group, which was confirmed in the laboratory findings of the current study by decreased MDA, NO, and cortisol serum levels and increased serum concentrations of GSH, TAC, and testosterone in the premix group as compared with the control group.

Thus, oxidative stress results when reactive substances, including reactive nitrogen, oxygen, and chlorine species, exceed the available antioxidant capability in the body. If the antioxidant systems in the animal body become incapable of adequately coping with enhanced reactive oxygen species (ROS) amounts and with the subsequent series of reactions of harmful macromolecule modifications (e.g., nucleic acids, proteins, and lipids) (54, 55), as a result of the stress factors, the hypothalamus liberates two neurohormones (corticotropin-releasing hormone (CRH) and arginine vasopressin), which are transported to induce the anterior pituitary gland to release ACTH, with subsequent release of the cortisol hormone “stress hormone” (56, 57). The stimulation of the hypothalamic–pituitary–adrenal (HPA) axis directly inhibits the hypothalamic–pituitary–gonadal (HPG) axis and Leydig cells in the testes. Thus, the main regulators of the HPA axis include CRH, AVP, glucocorticoids, and ACTH. The “stress system” affects male reproductive function negatively, where glucocorticoids affect testicular function at multiple levels of the HPG axis through their receptors in the hypothalamic neurons, pituitary gland, and testes (58).

Regarding the obtained data, there were significant reductions in NO and MDA. On the other hand, GSH and TAC concentrations were significantly elevated in the premix group in comparison to the control group; this could be due to the antioxidant effects of vitamins E, C, and A contained in the mineral-vitamin premix (59–61). Thus, supplementing camels with exogenous antioxidants, including vitamins E and C, leads to an improvement in the animal's antioxidant status and seems to be very helpful against ROS (62). In addition, in biological systems, vitamin E is considered a crucial antioxidant that acts as a robust chain-breaking factor by being a scavenger of peroxyl radicals and by cutting off the chain reaction of lipid peroxidation in membranes and lipoproteins (63).

Moreover, the presence of Se in the constituent of the mineral-vitamin premix acts as a structural element of the antioxidant glutathione peroxidase enzyme, which efficiently decreases lipid peroxides, H_2_O_2_, and phospholipid membrane oxidation which are essential for protection from oxidative stress (64). Furthermore, Se is necessarily required to maintain many metabolic functions and the growth performance of ruminants. Being an antioxidant element, it acts as a selenoprotein for the principal enzymes of the antioxidant defense mechanism, including glutathione peroxidase (GSH-Px) and thioredoxin reductase (TrxR) (65). More than that, Fe, Zn, Cu, and Mn are structural components of SODs, which are subdivided into four groups on the basis of their metal cofactors, including Fe-SOD, Cu/Zn-SOD, and Mn-SOD (66).

In this study, a significant decrease in cortisol levels was recorded in the premix group as compared with the control group. This finding could be due to vitamin C, which is one of the antioxidant constituents in the premix. Findings in this study match those who recorded a significant decrease in serum cortisol levels in broilers under heat stress and treated with vitamin C (55). In the same aspect, Stachowicz and Lebiedzińska (57) recorded that after acute psychological stress, a dose of 3,000 mg of vitamin C per day leads to faster cortisol recovery. In addition, Brody et al. (67) found that giving approximately 1,000 mg of vitamin C and 200 mg of vitamin E daily to elderly women suffering from coronary heart disease for 16 weeks led to a reduction in serum cortisol levels and an improvement in immune function compared with their own baselines. More than that, vitamin B6, vitamin B12, and folic acid accelerate the activity of γ-aminobutyric acid (GABAergic), which is a chemical compound that reduces the secretion of CRH (57, 68).

The testosterone hormone, which is considered the main sex hormone, has a controlling effect on reproductive functions such as libido, the last stages of spermatogenesis, and accessory sex gland activity in male animals (69). Our data revealed that testosterone levels had significantly increased in the premix group compared with the control. The obtained result could be due to the decreased level of the stress hormone (cortisol); those elevated levels inhibit testosterone synthesis (57).

In the current study, the results revealed a significant increase in T3 and T4 serum levels in the premix group in comparison with the control group. The obtained data could be due to the iodine supplement in the premix, where iodine is a rate-limiting component for T3 and T4 synthesis, as well as the single iodine physiological function in the human body, which is to synthesize T3 and T4 by the thyroid gland (70). In addition, camel dietary feeding programs need a very small percentage of iodine, which is necessary for appropriate animal function. Moreover, iodine plays a principal role in thyroid hormone formation, which participates in fetal brain development as well as regulating basic metabolism consumptions such as carbohydrates, fats, proteins, and heat formation processes (71) to affect reproductive, growth, and development functions (72).

In the present study, significant elevations were recorded in serum levels of Ca, P, and K in the premix group as compared with the control group. Minerals act as hormonal constituents of the endocrine system (73, 74). In addition, supplementing adequate quantities of essential minerals is essential to maintaining health status and the maximum productivity level of dairy cows (75). Moreover, electrolytes, trace elements, and steroid hormones play a significant role in the regulation and control of reproductive functions in both male and female animals. Furthermore, it is found that the blood testosterone concentrations in camels are significantly correlated with the contents of Mg, K, Na, and Ca in all genital organs, but only with the epididymal contents of P and Fe (76).

The blood glucose concentration in this study was significantly reduced in the premix group compared with the control group. The higher blood glucose level in the control group is associated with the stress due to rut season, which is accompanied by an elevated cortisol level, where cortisol stimulates hepatic glycogen stores, reducing glucose oxidation, activating lipolysis, and intensifying amino acid gluconeogenesis, as well as developing insulin resistance in cases of persistently high levels of this hormone accompanied by the mobilization of glucose reserves, which is specifically vital in cases of fast stress conditions due to extended effort (9, 47, 57).

On the other hand, a significant increase in TP and albumin was recorded in the premix group as compared with the control group, which could be due to the significant increase in feeding and rumination behavior (frequency and duration) between the premix group and the control group. Furthermore, it could be due to vitamin A, which is one of the components of the premix and is very important in many physiological functions, particularly reproduction, growth, differentiation of tissues, and maintenance of the epithelium. It has a natural antioxidant property and is necessary to maintain the integrity of the tissue lining the GIT and genitourinary tract, as well as to contribute to ensuring the optimal functioning of the immune system (77, 78).

## Conclusion

The rutting period compels a stressful condition in male camels, which is associated with elevated serum cortisol levels accompanied by decreased testosterone hormone concentration in the blood. Therefore, the feeding of minerals and vitamins to male camels during rut season is necessary to overcome the oxidative stress to which the animal is subjected, with a subsequent decrease in cortisol level followed by a decrease in aggressive behavior and an increase in testosterone hormone in the blood, in addition to a significant increase in feeding, rumination, and sexual behavior and an improvement in body condition score.

## Data availability statement

The original contributions presented in the study are included in the article/supplementary material, further inquiries can be directed to the corresponding authors.

## Ethics statement

The animal study was approved by the experimental protocol regarding the care and management of the camels was approved by the ZU-IACUC Committee under the number (ZU-IACUC\2\F\80\2023). The study was conducted in accordance with the local legislation and institutional requirements.

## Author contributions

NN, RE-S, and GA-R conceived, designed, performed the experiments, and analyzed the data. WS contributed to the analysis tools. NN, RE-S, HG, and WS wrote the manuscript. IR, FZ, and ZF revised and edited the manuscript. All authors have read and agreed to the published version of the manuscript.

## References

[1] El-Bahrawy KA Khalifa MA Rateb SA. Recent advances in dromedary camel reproduction: An Egyptian field experience. Emirates J Food Agric. (2015) 27:350–4. 10.9755/ejfa.v27i4.19907

[2] Mostafa TH Bakr AA Ayyat MS. Reproductive and Productive Efficiency of Maghrebi Dairy She-Camels Fed Diets Supplemented with Zinc-Methionine. Biol Trace Elem Res. (2020) 194:135–44. 10.1007/s12011-019-01744-0 31066019

[3] Abdelrahman MM Alhidary IA Aljumaah RS Faye B. Blood Trace Element Status in Camels: A Review. Animals. (2022) 12:2116. 10.3390/ani12162116 36009706 PMC9405446

[4] Padalino B Monaco D Lacalandra GM. Male camel behavior and breeding management strategies: How to handle a camel bull during the breeding season? Emir JFood Agric. (2015) 4:338–49. 10.9755/ejfa.v27i4.19909

[5] Marai IF Zeidan AE Abdel-Samee AM Abizaid A Fadiel A. Camels' reproductive and physiological performance Traits as affected by environmental conditions. Trop Subtrop Agroecosyst. (2009) 10:129–49. Available online at: https://www.redalyc.org/articulo.oa?id=93912989002

[6] Al-Juboori AT Mohammed M Rashid J Kurian J El-Refaey S Brebbia CA. Nutritional and medicinal value of camel (*Camelus dromedarius*) milk. In: Second International Conference on Food and Environment: The Quest for a Sustainable Future, Budapest, Hungary (2013). p. 221–232. 10.2495/FENV130201

[7] Skidmore JA. Reproductive Physiology in Male and Female Camels. In:Skidmore JA Adams GP., editors. International Veterinary Information Service (2000).

[8] Fatnassi M Padalino B Monaco D Khorchani T Lacalandra GM Hammadi M. Evaluation of sexual behavior of housed male camels (*Camelus dromedarius*) through female parades: correlation with climatic parameters. Trop Anim Health Prod. (2014) 46:313–21. 10.1007/s11250-013-0489-x 24122649

[9] Elias EM Weil S. Serum cortisol levels in camels (*Camelus dromedarius*) during the reproductive cycle. Comp Biochem Physiol A Comp Physiol. (1989) 94:787–90. 10.1016/0300-9629(89)90634-8 2575963

[10] El-Shoukary RD Nasreldin N Osman AS Hashem NM Saadeldin IM Swelum AA. Housing management of male dromedaries during the rut season: Effects of social contact between males and movement control on sexual behavior, blood metabolites and hormonal balance. Animals. (2020) 10:1–11. 10.3390/ani10091621 32927818 PMC7552277

[11] El shoukary R Osman A. Mitigates the negative behavioral effects of rut season in Camels: - Group Size. SVU-Int J Vet Sci. (2020) 51:253–262. 10.21608/svu.2019.17256.1031

[12] Larson C. Role of Trace Minerals in Animal Production. What do I need to know about trace minerals for beef and dairy cattle, horses, sheep and goats? Nutr Conf. (2005) 12:94. Available online at: http://animalrange.montana.edu/documents/courses/ANSC320/ConnieLarsenTraceminerals.pdf

[13] El-salaam A Bakr AA. Impact of dietary iodine supplementation on productive and reproductive performance of Maghrebian She-camels. IOSR-JAVS. (2018) 11:59–69. 10.9790/2380-1103015969

[14] Bach A. Key indicators for measuring dairy cow performance. In: Proceedings of the Proceedings of the FAO Symposium: Optimization of feed use efficiency in ruminant production systems. (2012). p. 33–44.

[15] El-Shoukary RD Batih GS Alkazmi L Amer HY Abou-Elnaga AF. Time-budget and blood parameters of the dromedary camels (*Camelus dromedarius*) under different feeding management strategies. Adv Anim Vet Sci. (2021) 9:895–903. 10.17582/journal.aavs/2021/9.6.895.903

[16] Bell C. Reproduction of the female camel (Camelus dromedarius and Camelus bacterianus). Hannover, Germany: Tierarztliche Bochschule Hannover (1999).16726319

[17] Faye B Bengoumi M Cleradin A Tabarani A Chilliard Y. Body condition score in dromedary camel: A tool for management of reproduction. Emirates J Food Agric. (2001) 13:1–6. 10.9755/ejfa.v12i1.5193

[18] Osman AM El Azab EA. Gonadal and epididymal sperm reserves in the camel, *Camelus dromedarius*. Reproduction. (1974) 38:425–30. 10.1530/jrf.0.0380425 4833818

[19] Dawkins MS. Observing Animal Behaviour: Design and Analysis of Quantitative Data. Oxford: Oxford University Press (2007).

[20] Stadler ES Santos LC Bertagnon HG Virmond MP Souza AM de Mizubuti IY. Performance of feedlot cattle with inclusion of live yeast in the diet. Semina: Ciências Agrárias. (2019) 40:2733. 10.5433/1679-0359.2019v40n6p2733

[21] Robinson I. PET-LIVESTOCK SOMALIA: A Pictorial Evaluation Tool (PET) for Livestock Condition Scoring in Somalia. Wales, UK: AA International Ltd: Aberystwyth (2010).

[22] Iglesias C Navas FJ Ciani E Arando A González A Marín C. Zoometric characterization and body condition score in Canarian camel breed. Arch Zootec. (2020) 69:14–21. 10.21071/az.v69i265.5034

[23] Faye B Bengoumi M Messad S Chilliard Y. Estimation des réser¬ves corporelles chez le dromadaire. Rev d'élevage Médecine Vétérinaire Trop. (2002) 55:69–78. 10.19182/remvt.9849

[24] Samanta S Sharma A Das B Mallick AK Kumar A. Significance of total protein, albumin, globulin, serum effusion albumin gradient and LDH in the differential diagnosis of pleural effusion secondary to tuberculosis and cancer. JCDR. (2016) 10:BC14. 10.7860/JCDR/2016/20652.8379 27656432 PMC5028544

[25] SPSS. SPSS user's guide statistics Version 10. Copyright SPSS Inc., USA (2001).

[26] Ghosh CP Datta S Mandal D Das AK Roy DC Roy A. Body condition scoring in goat: Impact and significance. J Entomol Zool Stud. (2019) 7:554–60. Available online at: https://www.entomoljournal.com/archives/2019/vol7issue2/PartJ/7-2-62-202.pdf

[27] Maurya VP Sejian V Kumar D Joshi A Naqvi SMK Karim SA. Body Condition Scoring system: A simple tool for optimizing productivity in sheep farms. Technical Bulletin, Central Sheep and Wool Research Institute, Avikanagar, Rajasthan. (2008).

[28] Khan BB Iqbal A Riaz M. Productionand Management of Camels. Faisalabad: University of Agriculture (2003).

[29] Faye B Bengoumi M Tressol JC. Compararative trace-element excretion in the camel and cow. J Camel Pract Res. (1999) 6:19–25.

[30] McDowell LR. Minerals in Animal and Human Nutrition. New York, NY: Elsevier Science BV (2003). 10.1016/B978-0-444-51367-0.50010-6

[31] Taneja SK Jain M Mandal R Megha K. Excessive zinc in diet induces leptin resistance in Wistar rat through increased uptake of nutrients at intestinal level. J Trace Elem Med Biol. (2012) 26:267–72. 10.1016/j.jtemb.2012.03.002 22683053

[32] Ebrahimi R. Effect of energy and protein levels on feedlot performance and carcass characteristics of Mehraban ram lambs. Pakistan J Biolog Sci. (2007) 10:1679–84. 10.3923/pjbs.2007.1679.1684 19086517

[33] Sahoo B Kumar R Garg AK Mohanta RK Agarwal A Sharma AK. Effect of supplementing area specific mineral mixture on productive performance of crossbred cows. Indian J Anim Nutr. (2017) 34:414–9. 10.5958/2231-6744.2017.00066.4

[34] Spears JW. Trace mineral bioavailability in ruminants. J Nutr. (2003) 133:1506S−9S. 10.1093/jn/133.5.1506S 12730454

[35] Hassan EH Farghaly MM Solouma GM. Effect of zinc supplementation from inorganic and organic sources on nutrient digestibility, some blood metabolites and growth performance of growing buffalo calves. Egypt J Nutr Feed. (2016) 19:37–46. 10.21608/ejnf.2016.74863

[36] Alhidary IA Abdelrahman MM Harron RM. Effects of a long-acting trace mineral rumen bolus supplement on growth performance, metabolic profiles, and trace mineral status of growing camels. Trop Anim Health Prod. (2016) 48:763–8. 10.1007/s11250-016-1022-9 26894497

[37] Puchala R Sahlu T Davis JJ. Effects of zinc-methionine on performance of Angora goats. Small Rumin Res. (1999) 33:1–8. 10.1016/S0921-4488(98)00194-1 10924874

[38] Hammadi M Khorchani T Khaldi G Majdoub A Abdouli H Slimane N. Effect of diet supplementation on growth and reproduction in camels under arid range conditions. Biotechnol Agron Soc Environ. (2001) 5:69–72. Available online at: https://popups.uliege.be/1780-4507/index.php?id=14855

[39] Spears JW Kegley EB. Effect of zinc source (zinc oxide vs zinc proteinate) and level on performance, carcass characteristics, and immune response of growing and finishing steers. J Anim Sci. (2002) 80:2747–52. 10.2527/2002.80102747x 12413098

[40] Richeson JT Kegley EB. Effect of supplemental trace minerals from injection on health and performance of highly stressed, newly received beef heifers. Prof Anim Sci. (2011) 27:461–6. 10.15232/S1080-7446(15)30519-2

[41] Faerevik G Andersen IL Jensen MB Bøe KE. Increased group size reduces conflicts and strengthens the preferences for familiar group mates after regrouping of weaned dairy calves (Bos taurus). Appl Anim Behav Sci. (2007) 108:215–28. 10.1016/j.applanim.2007.01.010

[42] Samimi AS. Aggressive sexual behavior of a dromedary bull causing sudden death in a male calf-camel. J Veter Behav. (2019) 31:1–4. 10.1016/j.jveb.2019.01.001

[43] Haley DB Rushen J Passillé AM. Behavioural indicators of cow comfort: Activity and resting behaviour of dairy cows in two types of housing. Can J Anim Sci. (2000) 80:257–63. 10.4141/A99-084

[44] Fregonesi JA Leaver JD. Behaviour, performance and health indicators of welfare for dairy cows housed in straw yard or cubicle systems. Livest Prod Sci. (2001) 68:205–16. 10.1016/S0301-6226(00)00234-7

[45] Hafez ESE. The Behaviour of Domestic Animals. 3rd ed. London: Bailliere, Tindall (1975). p. 203–245.

[46] Fink G. Encyclopedia of Stress. London: Academic Press (2000).

[47] Mohammed AAA Mohamed RD Osman A. Effects of stocking density on some behavioral and some blood biochemical parameters in camel during the rut period. Egypt J Vet Sci. (2020) 51:253–62. 10.21608/ejvs.2020.24526.1153

[48] McDonalds LE. Veterinary Endocrinology and Reproduction. 1st ed. Philadelphia, USA: Lea and Febiger. (1969).

[49] Burchfield SR Woods SC Elich MS. Pituitary adrenocortical response to chronic intermittent stress. Physiol Behav. (1980) 24:297–302. 10.1016/0031-9384(80)90090-6 6246558

[50] Stephens DB. Stress and its measurments in domestic animals. Adv Vet Comp Msd. (1981) 24:179–210. 7006340

[51] Hopster H van der Werf JT Erkens JH Blokhuis HJ. Effects of repeated jugular puncture on plasma cortisol concentrations in loose-housed dairy cows. J Anim Sci. (1999) 77:708–14. 10.2527/1999.773708x 10229368

[52] Sapolsky RM Romero LM Munck AU. How do glucocorticoids influence stress responses? Integrating permissive, suppressive, stimulatory, and preparative actions. Endocrine Rev. (2000) 21:55–89. 10.1210/edrv.21.1.0389 10696570

[53] Emeash HH Mostafa AS Karmy M Khalil F Elhussiny MZ. Assessment of transportation stress in Dromedary camel (*Camelus dromedarius*) by using behavioural and physiological measures. J Appl Veter Sci. (2016) 1:28–36. 10.21608/javs.2016.61827

[54] Lushchak VI. Free radicals, reactive oxygen species, oxidative stress and its classification. Chem Biol Interact. (2014) 224:164–75. 10.1016/j.cbi.2014.10.016 25452175

[55] del Barrio AS Mansilla WD Navarro-Villa A Mica JH Smeets JH den Hartog LA. Effect of mineral and vitamin C mix on growth performance and blood corticosterone concentrations in heat-stressed broilers. J Appl Poult Res. (2020) 29:23–33. 10.1016/j.japr.2019.11.001

[56] Buckingham JC. Glucocorticoids: exemplars of multitasking. Br J Pharmacol. (2006) 147:S258–68. 10.1038/sj.bjp.0706456 16402112 PMC1760726

[57] Stachowicz M Lebiedzińska A. The effect of diet components on the level of cortisol. Eur Food Res Technol. (2016) 242:2001–9. 10.1007/s00217-016-2772-3

[58] Nargund VH. Effects of psychological stress on male fertility. Nat Rev Urol. (2015) 12:373–82. 10.1038/nrurol.2015.112 26057063

[59] Abd Ellah MR Ghada AAE Abdel-Rady A. Relationship between antioxidants, nematode parasitic infestation of dromedary camels (*Calmelus dromedaries*) in Egypt. Assiut Vet Med J. (2008) 54:168–76. 10.21608/avmj.2008.175934

[60] Poljsak B Šuput D Milisav I. Achieving the balance between ROS and antioxidants: When to use the synthetic antioxidants. Oxid Med Cell Longev. (2013) 2013:956792. 10.1155/2013/956792 23738047 PMC3657405

[61] Pisoschi AM Pop A. The role of antioxidants in the chemistry of oxidative stress: A review. Eur J Med Chem. (2015) 97:55–74. 10.1016/j.ejmech.2015.04.040 25942353

[62] Chakir Y EL Khasmi M Farh M Bargaâ R Riad F Safwate A. Effects of vitamin E and vitamin C on hydrogen peroxide-induced hemolysis in Moroccan Dromedary camels (*Camelus dromedarius*). Greener J Med Sci. (2013) 3:111–20. 10.15580/GJMS.2013.4.032713548

[63] Shirpoor A Khadem AM Salami S Ghaderi PF Rasmi Y. Effect of vitamin E on oxidative stress status in small intestine of diabetic rat. World J Gastroenterol. (2007) 13:4340–4. 10.3748/wjg.v13.i32.4340 17708608 PMC4250861

[64] Shahin MA Khalil WA Saadeldin IM Swelum AA El-Harairy MA. Effects of vitamin C, vitamin E, selenium, zinc, or their nanoparticles on camel epididymal spermatozoa stored at 4°C. Trop Anim Health Prod. (2021) 53:1–9. 10.1007/s11250-020-02521-1 33411090

[65] Khalil MMH Soltan YA Khadiga GA Elmahdy A Sallam SMA Zommara MA. Comparison of dietary supplementation of sodium selenite and bio-nanostructured selenium on nutrient digestibility, blood metabolites, antioxidant status, milk production, and lamb performance of Barki ewes. Animal Feed Sci Technol. (2023) 297:115592. 10.1016/j.anifeedsci.2023.115592

[66] Pedone E Fiorentino G Bartolucci S Limauro D. Enzymatic antioxidant signatures in hyperthermophilic archaea. Antioxidants. (2020) 9:1–19. 10.3390/antiox9080703 32756530 PMC7465337

[67] Brody S Preut R Schommer K Schürmeyer TH A. randomized controlled trial of high dose ascorbic acid for reduction of blood pressure, cortisol, and subjective responses to psychological stress. Psychopharmacol (Berl). (2002) 159:319–24. 10.1007/s00213-001-0929-6 11862365

[68] Miklos IH Kovacs KJ. GABAergic innervation of corticotropin-releasing hormone (CRH)-secreting parvocellular neurons and its plasticity as demonstrated by quantitative immunoelectron microscopy. Neuroscience. (2002) 113:581–92. 10.1016/S0306-4522(02)00147-1 12150778

[69] Hafez ES Hafez B. Reproductive Cycles. Reproduction in Farm Animals. Philadelphia, PA: Lippincott Williams & Wilkins (2000). p. 55–67. 10.1002/9781119265306.ch4

[70] Chung HR. Iodine and thyroid function. Ann Pediatr Endocrinol Metab. (2014) 19:8–12. 10.6065/apem.2014.19.1.8 24926457 PMC4049553

[71] Cann SA. Hypothesis: dietary iodine intake in the etiology of cardiovascular disease. J Am Coll Nutr. (2006) 25:1–11. 10.1080/07315724.2006.10719508 16522926

[72] Georgievskii VI Annenkov BN Samokhin VT. Mineral Nutrition of Animals: Studies in the Agricultural and Food Sciences. Oxford: Elsevier. (2013).

[73] Suttle NF. Mineral nutrition of livestock. Cabi. (2010). 10.1079/9781845934729.0000

[74] Costa RM Ponsano EH de Souza VC Malafaia P. Reduction of phosphorus concentration in mineral supplement on fertility rate, maternal ability and costs of beef cows reared in pastures of Urochloa decumbens. Trop Anim Health Prod. (2016) 48:417–22. 10.1007/s11250-015-0967-4 26685846

[75] Council NR. Nutrient Requirements of Dairy Cattle. London: National Academies Press (2001). 38386771

[76] Al-Qarawi AA Abdel-Rahman HA El-Belely MS El-Mougy SA. Age-related changes in plasma testosterone concentrations and genital organs content of bulk and trace elements in the male dromedary camel. Anim Reprod Sci. (2000) 62:297–307. 10.1016/S0378-4320(00)00146-9 10924832

[77] Vasudevan DM Sreekumari S. Textbook of Biochemistry for Medical Students. 3rd ed. New Delhi: JB Medical Ltd. (2001).

[78] Lal H Pandey R. A Textbook of Biochemistry. New Delhi: CBS Publishers (2004).

